# Genome sequence of multi-drug-resistant *Staphyloccocus aureus* isolated from a clinical sample collected in Maroua, Cameroon

**DOI:** 10.1128/mra.00656-24

**Published:** 2024-09-30

**Authors:** Mansour Mohamadou, Emmanuel Kagning Tsinda, Qudsia Hina, Kashaf Javed, Rodrigue Roman Dongang Nana, Marie Chantal Ngonde Essome, Sara Riwom Essama, Sadia Sattar, Hortense Gonsu Kamga, Sundus Javed

**Affiliations:** 1 Centre for Research on Health and Priority Pathologies, Institute of Medical Research and Medicinal Plants Studies, Yaoundé, Cameroon; 2 Biosciences Department, COMSATS University Islamabad, Islamabad, Pakistan; 3 Department of Microbiology, Faculty of Science, University of Yaounde, Yaoundé, Cameroon; 4 Center for Biomedical Innovation, Massachusetts Institute of Technology, Cambridge, Massachusetts, USA; 5 Department of Microbiology, Hematology and Infectious Diseases, Faculty of Medicine and Biomedical Sciences, University of Yaounde, Yaoundé, Cameroon; Loyola University Chicago, Chicago, Illinois, USA

**Keywords:** genomes, multidrug resistance, *Staphylococcus aureus*, next-generation sequencing

## Abstract

*Staphylococcus aureus* is a major pathogen of public health concern due to its implications in pathologies and its increasing antimicrobial resistance. Here, we present the draft genome of a 2.7-Mbp *S. aureus* isolate obtained from a pus swab sampled in Cameroon. The GC content of the draft genome is 32%.

## ANNOUNCEMENT

*Staphylococcus aureus* is a Gram-positive bacteria causing a wide range of infections ranging from skin infections to life-threatening pneumonia, sepsis, food poisoning, and endocarditis (1, 2). Antibiotic resistance poses a major threat to human health around the world, and recent analysis of *S. aureus* genomes in northern Cameroon found a 53% prevalence of multi-drug-resistant *S. aureus* strains (3, 4). In this study, we determined the draft genome sequence from a clinical isolate of *S. aureus* E2-4 strain detected in Northern Cameroon.

Ethics approval (number 2017/12/958/CE/CNERSH/SP) was received before the study. Pus swabs were collected from a 42-year-old patient at the clinical laboratory of Maroua Regional Hospital (10.5925 N 14.3210 E). Bacterial culture was performed using Mannitol salt Agar (Bio-Rad, France) incubated at 37°C for 24 h under aerobic conditions, and identification was performed through colony morphology observation, in this case the colonies of *S. aureus* which appeared large, round, and golden yellow. Then Gram staining, DNase, coagulase, and catalase tests were performed. PCR was used to confirm *S. aureus* identification followed by antimicrobial susceptibly testing performed as described earlier (3).

A Mannitol Salt plate with colonies was shipped to Macrogen (Seoul, Korea) for sequencing. At Macrogen, DNA was extracted from selected colonies by harvesting cells from agar plate in 200 µL of 10 mM TE buffer and thorough vortexing. For DNA extraction, the boiling method, utilizing Instagene matrix, was employed, and sequencing libraries were prepared using the Nextera XT DNA Library Prep Kit. Next-generation sequencing was performed using the Illumina MiSeq platform, generating a total of 12,892,818 pairs of 75 bp reads. Bioinformatic analyses were performed using packages running within BV-BRC 3.37.14, using default parameters (5). In brief, data quality control was done using FastQC v 0.11.9 (6). Reads were trimmed Trim galore v0.0.5, then *de-novo* assembled using Unicycler v0.4.8 and polished using Pilon v1.23 (7–9). Samtools v1.17 generated alignment statistics, and the genome was annotated using PGAP v5.2 (10, 11).

The draft genome of *S. aureus* E2-4 was rated as good quality by BV-BRC 3.37.14 because of the genome’s 100% completeness, 0.2% contamination, a coarse consistency of 100%, and a fine consistency of 99.5%. *S. aureus subspecie aureus* NCTC 8325 (Accession #CP000253) showed the highest genome homology to *S. aureus* E2-4 using the similar Genome Finder tool in BV-BRC 3.37.14. The estimated GC content of 32.69% and the length of 2,750,684 bp were similar to previously reported statistics for antibiotic-resistant *S. aureus* strains (12, 13). *De novo* assembly yielded a total of 43 contigs. The contig length ranged from 383 bp to 417,195 bp, with an N50 of 180,319 bp and L50 of 5. The average coverage depth was 690×. The annotated genome displays 2,598 protein-coding sequences, 52 transfer RNA genes, and 4 ribosomal RNA genes.

ResFinder v4.5.0 identified BlaZ, tet(K), and dfrG genes which likely contribute to the multi-drug resistance phenotype (Table 1) (14).

**TABLE 1:** List of antibiotic resistance genes identified and the corresponding resistance mechanism

	Antibiotic resistance genes			
Variables	blaZ	tet(K)	tet(K)	dfrG
Identity (%)	100	99.89	100	100
Alignment length/gene length	888/888	958/1,380	422/1380	498/498
Breadth of coverage (%)	100	69.42	30.57	100
Reference accession number	CP003970	U38656	U38656	AB205645
Position in reference	1..888	423..1380	1..422	1..498
Contig number	assembly_contig_5	assembly_contig_30	assembly_contig_30	assembly_contig_23
Position in contig	71,417..72,304	1..958	4,018..4,439	17,530..18,027
Phenotype	Amoxicillin, Ampicillin, Penicillin, Piperacillin	Doxycycline, Tetracycline	Doxycycline, Tetracycline	Trimethoprim

## Data Availability

The whole genome of E2-4 can be found in GenBank accession number JBEGCM000000000. The associated BioProject and BioSample accession numbers are PRJNA1111884 and SAMN41405411, respectively.
